# Diagnosis of Spontaneous Bacterial Peritonitis and an *In Situ* Hybridization Approach to Detect an “Unidentified” Pathogen

**DOI:** 10.1155/2014/634617

**Published:** 2014-07-15

**Authors:** Hirayuki Enomoto, Shin-ichi Inoue, Akio Matsuhisa, Shuhei Nishiguchi

**Affiliations:** ^1^Division of Hepatobiliary and Pancreatic Disease, Department of Internal Medicine, Hyogo College of Medicine, Mukogawacho 1-1, Nishinomiya, Hyogo 663-8501, Japan; ^2^Research & Development Center, Fuso Pharmaceutical Industries, Ltd., Morinomiya 2-3-30, Joto-ku, Osaka 536-8523, Japan

## Abstract

Spontaneous bacterial peritonitis (SBP) is a frequent and severe complication in cirrhotic patients with ascites. Although identifying the pathogen(s) plays a major role in the management of infectious diseases, ascitic fluid cultures often show negative results in patients with clinical signs and symptoms of SBP, and ascitic fluid cell analyses are the gold standard method for diagnosing SBP. SBP is generally diagnosed based on an increased number of polymorphonuclear neutrophils in the ascitic fluid (>250/mm^3^), and the identification of the causal pathogen may not be given consideration. We newly developed an *in situ* hybridization (ISH) method to provide early and direct evidence of bacterial infection in ascites in patients with SBP. This paper will review the diagnosis of SBP, including our novel approach with ISH method to detect bacterial DNA in SBP ascitic fluid.

## 1. Introduction

Spontaneous bacterial peritonitis (SBP) is a frequent and severe complication in patients with cirrhosis and ascites. SBP is a bacterial infection that occurs in the absence of an evident intra-abdominal and surgically treatable source of infection, such as the perforation or inflammation of intra-abdominal organs [1–4]. Although the precise mechanism(s) underlying the development of SBP have not been fully clarified, bacterial translocation (BT) is believed to be the most important causative factor. Mild BT to the mesenteric lymph nodes is a documented physiological event; however, only a few intestinal bacteria, including* Escherichia coli*,* Klebsiella pneumoniae*, and other Enterobacteriaceae, are able to efficiently translocate from the lumen of the gut to the mesenteric lymph nodes [5, 6]. Since the bacterial species with a capacity for BT are also major pathogens of SBP, unphysiological disease-related BT is thought to be significantly associated with the development of SBP. In addition, several conditions frequently noted in cirrhotic patients, including alterations in gut flora, increased intestinal permeability, and a compromised immune system, have been reported to be involved in disease-related BT and the subsequent onset of SBP [6].

The prevalence of SBP in cirrhotic hospitalized patients with ascites ranges from 10% to 30% [1, 2, 7]. Although the mortality rate was initially reported to exceed 90%, the prognosis has improved with early diagnosis and treatment [8]. The diagnosis of SBP is established based on positive ascitic fluid bacterial cultures and the detection of an elevated absolute fluid polymorphonuclear neutrophil (PMN) count in the ascites (>250/mm^3^) without an evident intra-abdominal surgically treatable source of infection [1, 9]. Although identifying the pathogen(s) plays a major role in the management of infectious diseases, it takes several days to identify the casual bacteria. In addition, ascitic fluid cultures are negative in approximately 10–60% of patients with clinical manifestations of SBP [3, 9, 10]. Therefore, SBP is usually diagnosed based only on an increased (>250/mm^3^) PMN count in the ascitic fluid in cases in which there is no obvious source of bacteria spreading to the ascites, regardless of whether ascitic fluid cultures are positive [6, 11–13]. This paper reviews the diagnosis of SBP, focusing on a novel* in situ* hybridization (ISH) method for detecting bacterial DNA in the ascites of patients with SBP.

## 2. Standard Approach to Diagnosing SBP

### 2.1. Diagnostic Paracentesis

Paracentesis is extremely important, as the PMN count in the ascitic fluid plays an essential role in obtaining a diagnosis of SBP. Diagnostic paracentesis should be performed in all patients who present with (1) compatible signs or symptoms (abdominal pain and/or tenderness on palpation, fever, and chills); (2) impairment of the hepatic or renal function; (3) unexplained hepatic encephalopathy; (4) gastrointestinal bleeding [6, 9, 11–13]. However, clinical signs and symptoms are occasionally absent in patients with SBP [11–16]. Although all cirrhotic patients with ascites are at risk of SBP, the prevalence of SBP among hospitalized patients (10%) is higher than that observed in outpatients (1.5–3.5%) [14, 15]. It is therefore recommended that diagnostic paracentesis be performed in all cirrhotic patients with ascites who require hospital admission, regardless of whether they exhibit clinical symptom(s) of SBP [1, 6, 9, 11–13].

### 2.2. Ascitic Fluid Cell Analysis

Despite the use of a sensitive method (the culture-bottle method; please refer to the Ascitic Fluid Culture section), ascites cultures often show negative results, even in patients with an increased ascitic PMN count and clinical symptoms suggestive of SBP [3, 9, 10, 18]. Therefore, the diagnosis of SBP is confirmed based on a PMN count in the ascites of >250 cells/mm^3^ in the absence of an intra-abdominal and surgically treatable source of infection. The cutoff value of 250 PMN cells/mm^3^ has the greatest sensitivity, whereas 500 PMN cells/mm^3^ exhibits the greatest specificity [19–21]. However, the most sensitive cutoff value should be used for diagnosis, as it is important not to miss cases of SBP. Physicians should subtract one PMN for every 250 red blood cells in patients with hemorrhagic ascites with a fluid red blood cell count of >10,000/mm^3^ (due to the effects of concomitant malignancy or traumatic tap) in order to adjust for the presence of blood in the ascites [9, 11–13].

The PMN count in the ascitic fluid may be determined according to a hematological method using either a light microscope and manual counting chamber or an automated cell counter [22–24]. The ascitic fluid is centrifuged in order to manual count the number of ascitic cells, after which a smear of the collected cells is stained with Giemsa and the total and differential cell counts are determined using a light microscope [1, 4, 25]. The microscopic cell counting method requires several hours and carries a risk at inter- and/or intraobserver discrepancy. On the other hand, automated cell counters provide reproducible results within a few minutes; however, coulter counter findings of the neutrophil count have been shown to be inaccurate for relatively low levels of neutrophils in the ascitic fluid. Therefore, the manual PMN counting method is conventionally preferred [1, 6]. However, a recent study demonstrated that automated cell counts have sufficient sensitivity for diagnosing SBP [22], thus suggesting that this simple method may be used in place of traditional manual counting.

### 2.3. Ascitic Fluid Culture

Conventional bacterial culture methods, such as laboratory analyses of fluid collected in syringes or tubes, effectively detect bacteria in less than 50% of ascites samples with an elevated PMN count (>250/mm^3^). Therefore, it is recommended to inoculate the ascitic fluid into blood culture bottles at the patient's bedside in order to increase the sensitivity of the bacterial culture [10, 26–29]. The culture-positive rate of SBP ascites is approximately 80%, namely, between 72% and 90% of cases assessed using the culture-bottle method [9, 11]. However, several recent studies have reported lower culture-positive rates for SBP ascites, ranging from approximately 40% to 60% [3, 30–32]. In addition, even with the sensitive culture-bottle method, positive results for ascitic cultures are estimated to be approximately 40–70%, according to various recent guidelines [6, 11–13]. Since patients with an increased PMN count in the ascitic fluid (>250 cells/mm^3^) and negative cultures exhibit a clinical presentation similar to that of bacteriologically confirmed SBP [1, 33], these patients are categorized as having “culture-negative SBP” and should be treated in the same manner as those with culture-positive SBP.

### 2.4. Differentiation from Secondary Bacterial Peritonitis

Differentiating SBP from secondary peritonitis due to perforation or inflammation of intra-abdominal organs is clinically very important. Secondary bacterial peritonitis should be suspected in patients with relevant abdominal signs or symptoms, multiple organisms in ascitic cultures, and a very high PMN count and/or high protein concentration in the ascites, as well as those who display an inadequate response to therapy [25]. However, accurately diagnosing secondary peritonitis based on these criteria generally takes a long time, and patients with perforated secondary peritonitis require surgical treatment in a timely fashion [34]. Therefore, performing abdominal CT to detect perforation is recommended in patients with suspected secondary bacterial peritonitis [6, 9, 11–13].

Various parameters available at the time of paracentesis have been proposed to assist in rapidly detecting secondary peritonitis. Parameters in the ascitic fluid in patients with secondary peritonitis, as proposed by Runyon and Hoefs [35], are as follows: (1) an elevated PMN count in the ascitic fluid (>250/mm^3^: usually many thousands) and (2) at least two of the following: a total protein level of >1 g/dL, a serum lactate dehydrogenase level above the upper limit of normal, and a glucose level of <50 mg/dL. In addition, both an alkaline phosphatase level of >240 U/l and carcinoembryonic antigen level of >5 ng/mL in the ascitic fluid have been reported to exhibit good diagnostic performance for detecting gut perforation into the ascitic fluid, with a sensitivity of 92% and specificity of 88% [30]. However, it is not easy to differentiate SBP from secondary peritonitis based only on biochemical parameters of ascitic samples, and abdominal CT is essential in the clinical setting [6, 11–13, 34, 35].

## 3. Potential Diagnostic Methods for SBP

### 3.1. Leukocyte Esterase Reagent Strips (LERS)

It takes several hours to obtain the results of an ascitic fluid cell count. Therefore, the use of leukocyte reagent strips (LERS) has been proposed as a fast and inexpensive method for diagnosing SBP. These reagent strips, which were originally developed to diagnose urinary tract infections, detect leukocytes based on their esterase activity according to a colorimetric method [36]. However, a large, multicenter prospective study recently showed that the Multistix 8 SG has a low level of diagnostic accuracy for diagnosing SBP, with a high false-negative rate (55%) [14]. In addition, a systemic review of 19 studies of several strips (including Multistix, Aution, Combur, Nephur, and UriScan) demonstrated that these LERS have both low sensitivity and a high risk of false-negative results [36]. According to a recent review of 26 studies regarding the validity of LERS for SBP diagnosis [37], LERS display low sensitivity for diagnosing SBP, with significant interstudy variability among brands of LERS. However, LERS have consistently shown high negative predictive value (>95% in the majority of studies) and may therefore be used as a preliminary screening tool to diagnose SBP. However, the utility of LERS for diagnosing SBP has not been confirmed.

Most of the above strips were developed for use in urine with a threshold of >50 PMN cells/mm^3^; however, the diagnostic performance of a reagent strip test calibrated for ascitic fluid with a cutoff of 250 PMN cells/mm^3^ has recently been reported [38]. That study showed excellent results, with a sensitivity of 100% and a negative predictive value of 100%. Although these conclusions have yet to be confirmed in large multicenter trials, this method may provide a new and useful diagnostic tool for detecting SBP.

### 3.2. Measurement of Leukocyte-Derived Proteins

The levels of proteins, such as granulocyte elastase [39] and lactoferrin [40], released by activated PMNs are elevated in patients with SBP. Lactoferrin shows notable sensitivity (95.5%) and specificity (97%) for diagnosing SBP, with a cutoff value of 242 ng/mL [40]. Nevertheless, the diagnostic performance of this parameter must be further evaluated in other studies with a larger number of patients due to the small number of SBP cases in that study. In addition to the proteins described above, the levels of several inflammatory cytokines and chemokines in the ascitic fluid are reported to be associated with the severity of SBP [41, 42]. However, these potential diagnostic biomarkers are generated by host reactions against inflammatory stimulation and fail to provide any direct evidence of bacterial infection in SBP ascites.

### 3.3. Detection of Bacterial DNA Using Polymerase Chain Reaction (PCR)

Bacterial cultures require several days to obtain results. Hence, bacterial DNA detection and sequencing is increasingly being used to diagnose various infectious diseases [43–45]. Some PCR-based methods for detecting bacterial DNA have also been applied to the microbiological diagnosis of SBP [46–49]. However, these methods have received several major criticisms regarding the detection of bacterial DNA. First, most previous studies enrolled a limited number of patients, and a recent report including a large number of patients showed poor results for diagnosis. Furthermore, previous studies have revealed serious concerns regarding contamination of bacterial DNA in the PCR system [50–53]. Commercially available Taq-polymerases may be contaminated with bacterial DNA [50, 51]. Moreover, the reagents used for DNA extraction procedures carry a risk of exposing the clinical samples to exogenous bacterial DNA [52, 53]. Although PCR is a very sensitive method for detecting DNA, PCR-based methods display discrepant and controversial findings with respect to diagnostic performance in detecting the causative pathogen(s) in SBP patients with ascites [46–49], perhaps, or at least in part, due to the problems described above. Therefore, no definitive PCR-based method for providing an accurate diagnosis of SBP has been established.

## 4. A Novel Approach for Detecting Bacterial DNA in SBP Ascites Using* In Situ* Hybridization

A new strategy using an ISH method for detecting the genomic DNA of bacteria phagocytized in neutrophils and macrophages was recently developed to identify causal bacteria in cases of sepsis [54–56]. The utility of this ISH method for detecting bacterial genomic DNA phagocytized in the leukocytes of patients with sepsis has been demonstrated, providing evidence for the presence of bacterial infection in such cases. Notably, the ISH method is almost four times more sensitive than blood cultures in detecting the causal bacteria of sepsis [55]. In addition, the results of ISH tests can be acquired within one day, whereas it takes several days, at least, to obtain the results of cultures. Based on the rapid and sensitive detection of bacterial DNA provided by the ISH method, we investigated whether this method can be used to obtain direct evidence of bacterial infection in SBP patients with ascites [17].

**Figure 1:**
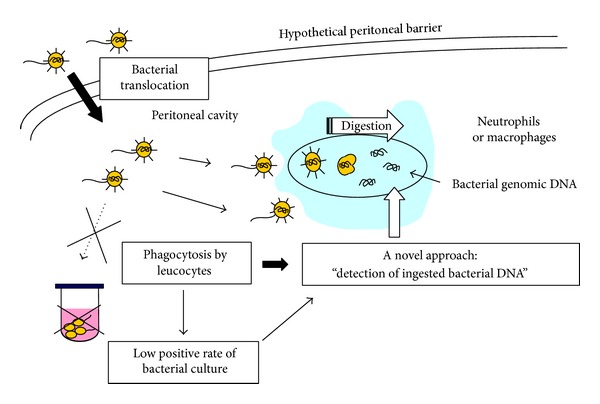
The concept of the ISH method for detecting bacterial DNA in SBP ascites.

The concept of the ISH method for detecting bacterial DNA in SBP patients with ascites is shown in Figure 1. In addition to the low amount of bacteria present in the ascitic fluid of SBP patients, phagocytosis and the digestion of bacteria by leukocytes may reduce the amount of proliferative, suspended bacteria in the ascitic fluid, thus making it difficult to identify the pathogen using standard methods. Phagocytosis is thought be responsible for the low rate of detectable causative bacteria [17]. Therefore, we attempted to detect ingested bacterial DNA using the ISH method. Since all bacteria have the 23S ribosomal RNA gene, a novel cDNA probe for this gene was generated to detect the genomic DNA of the causative bacteria.

**Figure 2:**
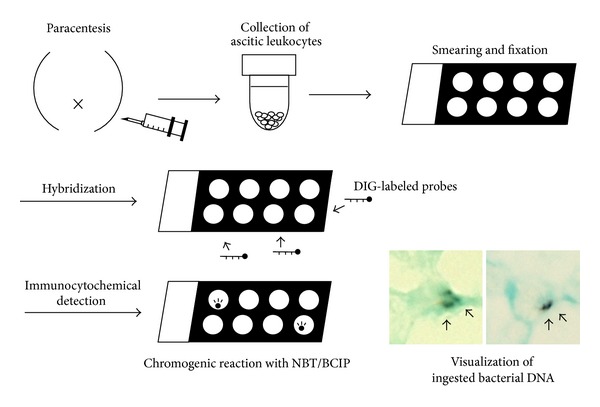
Schematic representation of the* in situ* hybridization (ISH) method used to assess the ascitic samples. Floating leukocytes in the ascitic fluid were collected via centrifugation and used for the ISH tests. DIG- (digoxigenin-) labeled probes were used for hybridization, and positive signals were detected with NBT (nitro-blue tetrazolium chloride) and BCIP (5-bromo-4-chloro-3′-indolyphosphate p-toluidine salt). Positive (purple brown) signals were observed in the leukocytes with intracellular bacterial DNA (arrows).

Several cDNA fragments corresponding to the 23S rRNA genes of various bacteria were obtained using PCR. Since we found no single cDNA probe able to detect all types of bacteria universally, we mixed plural cDNA fragments to create a new probe cocktail. This cocktail was able to detect the genomic DNA of all 59 bacterial strains examined, including the leading species accounting for SBP (Table 1). The new probe was designated the “global bacteria (GB) probe,” and its utility for detecting phagocytized bacterial DNA in SBP ascites was evaluated. An outline of the ISH method for assessing leukocytes in the ascitic fluid is shown in Figure 2. Floating leukocytes in the ascites were collected via centrifugation and prepared for the ISH tests. Intracellular bacterial DNA was detected as positive (purple brown) signals of leukocytes in the SBP samples.

The ISH tests showed positive results in 10 of 11 SBP ascites samples, whereas negative results were obtained in all remaining 40 non-SBP ascites samples. These findings suggest that the ISH test yields high sensitivity (91%) and specificity (100%) for detecting phagocytized bacterial DNA in leukocytes the ascites of SBP patients. Importantly, the ISH tests showed positive findings in seven cases with negative culture results, thus suggesting that the ISH method can be used to identify bacterial infections that are not detected using the bacterial culture method [17]. Furthermore, the ISH test results were obtained within one day, consistent with that observed for septic blood samples. Therefore, this newly established ISH method resulted in the rapid and sensitive detection of bacterial DNA in SBP ascites, thereby implying its utility for providing early and direct evidence of bacterial infection. Although additional large-scale clinical trials are required to evaluate the ISH method in detail, this new test may offer a novel and effective approach for the management of SBP.

## 5. Conclusion

Rapid diagnosis and treatment play a key role in the management of SBP. In general, potentially fatal cirrhosis-related infectious disease is diagnosed based only on an increased PMN count in the ascitic fluid, and the identification of the causal pathogen is sometimes not taken into consideration. Although no ideal method for detecting causal bacteria has been established, our novel ISH test may be used to provide early and direct evidence of bacterial infection in SBP patients with ascites. The current findings therefore shed new light on the management of SBP.

## Figures and Tables

**Table 1:** Bacterial strains detected by the *in  situ* hybridization method [17]. ISH test was able to detect the genomic DNA of all bacterial strains tested.

Genus	Species
*Eggerthella *	*lenta *
*Corynebacterium *	*diphtheriae *
	*pseudodiphteriticum *
	*jeikeium *
*Propionibacterium *	*acnes *
*Micrococcus *	*luteus *
*Lactobacillus *	*fermentum *
	*acidophilus *
*Bacillus *	*cereus *
*Staphylococcus *	*aureus *
	*epidermidis *
*Enterococcus *	*faecalis *
	*faecium *
	*avium *
*Streptococcus *	*pneumoniae *
	*sanguinis *
	*pyogenes *
	*agalactiae *
	*salivarius *
*Clostridium *	*perfringens *
*Peptoniphilus *	*asaccharolyticus *
*Bacteroides *	*fragilis *
	*ovatus *
*Porphyromonas *	*asaccharolytica *
*Fusobacterium *	*nucleatum *
	*necrophorum *
*Brevundimonas *	*diminuta *
*Burkholderia *	*cepacia *
*Achromobacter *	*xylosoxidans *
*Pseudomonas *	*aeruginosa *
	*fluorescens *
	*putida *
*Acinetobacter *	*calcoaceticus *
*Escherichia *	*coli *
*Enterobacter *	*cloacae *
	*sakazakii *
	*aerogenes *
	*gergoviae *
*Klebsiella *	*pneumoniae *
	*aerogenes *
	*oxytoca *
*Raoultella *	*terrigena *
*Haemophilus *	*influenzae *
*Serratia *	*marcescens *
	*liquefaciens *
*Citrobacter *	*koseri *
*Hafnia *	*alvei *
*Edwardsiella *	*tarda *
*Proteus *	*vulgaris *
	*mirabilis *
*Providencia *	*rettgeri *
	*alcalifaciens *
	*stuartii *
*Morganella *	*morganii *
*Salmonella *	*enterica *
*Pantoea *	*agglomerans *
*Kluyvera *	*intermedia *
*Raoultella *	*planticola *
*Stenotrophomonas *	*maltophilia *

## Abbreviations

SBP: Spontaneous bacterial peritonitis
BT: Bacterial translocation
PMN: Polymorphonuclear neutrophil
ISH: * In situ* hybridization
PCR: Polymerase chain reaction.
